# Structuring of Dragonfly Communities (Insecta: Odonata) in Eastern Amazon: Effects of Environmental and Spatial Factors in Preserved and Altered Streams

**DOI:** 10.3390/insects10100322

**Published:** 2019-09-27

**Authors:** José Max Barbosa Oliveira-Junior, Leandro Juen

**Affiliations:** 1 Programa de Pós-Graduação em Zoologia, Programa de Pós-Graduação em Ecologia, Laboratório de Ecologia e Conservação, Universidade Federal do Pará, Rua Augusto Correia, Nº 1, Bairro Guamá, Belém 66075-110, Pará, Brazil; leandrojuen@gmail.com; 2 Instituto de Ciências e Tecnologia das Águas, Universidade Federal do Oeste do Pará, Rua Vera Paz, s/n (Unidade Tapajós) Bairro Salé, Santarém 68040-255, Pará, Brazil

**Keywords:** Metacommunity, partitioning of variance, Anisoptera, Zygoptera, neutral theory, niche theory

## Abstract

The evaluation of the effects of environmental factors on natural communities has been one of the principal approaches in ecology; although, over the past decade, increasing importance has been given to spatial factors. In this context, we evaluated the relative importance of environmental and spatial factors for the structuring of the local odonate communities in preserved and altered streams. Adult Odonata were sampled in 98 streams in eastern Amazonia, Brazil. The physical features of each stream were evaluated and spatial variables were generated. Only environmental factors accounted for the variation in the Odonata community. The same pattern was observed in the suborder Zygoptera. For Anisoptera, environmental factors alone affect the variation in the community, considering all the environments together, and the altered areas on their own. As the two Odonata suborders presented distinct responses to environmental factors, this partitioning may contribute to an improvement in the precision of studies in biomonitoring. We thus suggest that studies would have a greater explanatory potential if additional variables are included, related to biotic interactions (e.g., competition). This will require further investigation on a finer scale of environmental variation to determine how the Odonata fauna of Amazonian streams behaves under this analytical perspective.

## 1. Introduction

One of the primary objectives of ecological research has traditionally been the understanding of the factors that determine the distribution of species in natural environments [1]. The variation over time in the composition and abundance of species in a community provides an important parameter for the understanding of the processes that determine its structure [1]. Local and regional processes, such as biotic and abiotic factors, the climate, primary productivity, habitat type, and interspecific interactions, all have a profound effect on community structure [2].

More complex environments would be expected to have a higher species richness due to the greater diversity of ecological niches, related primarily to the availability of feeding resources and environmental conditions [3]. From this perspective, most ecological studies have focused on the environmental factors that determine community structure on a local scale [4]. Most of these studies are based on the assumption that the availability of niches will determine the composition and diversity of a community, i.e., niche theory [5]. In this case, the distribution of species will be determined by abiotic factors and the way in which different species, with distinct requirements in terms of conditions and resources, will partition the existing niches, so that the greater the environmental heterogeneity, the higher the rate of species substitutions, and the lower the similarity among sites [5].

There is a contrasting spatial approach (neutral theory), however, which assumes that all species have identical aptitudes [6] and thus the same probability of colonizing any given environment. In this case, the principal factor driving the similarity of communities at a regional level would be the limitations imposed on dispersal by the geographic distance between communities [6,7]. In this case, sites located more closely together will be more likely to be colonized by members of the same species found in neighboring communities and, consequently, to share more species [4]. Even in completely homogeneous environments, then, dissimilarities among communities will arise as a result of the spatial limitations imposed by the dispersal capacity of the different species [6,8].

While studies of the structuring of natural communities have tended to focus on the role of local environmental conditions, such as disturbances, competition, and abiotic conditions [9], in recent years, spatial processes (geographic distance) have gained growing importance in the analysis of the influence of these different factors [10,11]. Countless studies have attempted to describe or account for the influence of environmental processes and/or spatial factors on the structure of dragonfly communities in various parts of the world [12,13,14,15,16,17,18], although only a relatively small number have focused explicitly on the contributions of both factors in the context of a single system [19,20]. Four notable exceptions, all from Brazil, are Juen and De Marco [4], Brasil [21], and Alves-Martins [22] with studies of adult Odonata in Amazonian streams, and the study of Mendes et al. [23], who focused on larvae in streams in the Amazon–Cerrado transition zone.

Major environmental disturbances may modify environmental and/or spatial factors, leading to the elimination of the most sensitive local species, and alterations in the organization of the community [24]. A marked reduction in the environmental integrity of a body of water may lead to modifications of the natural dynamics of biological communities, resulting in the loss of aquatic biodiversity [25,26]. Alterations to habitat structure caused by deforestation in the drainage basin affect trophic dynamics and may alter Odonata species composition and diversity [14,27,28,29,30,31,32,33]. As the distribution of many organisms is related directly to the effects of impacts on the ecosystem [34], it is fundamentally important to understand the respective effects of environmental and/or spatial factors in preserved and altered environments.

The order Odonata is amongst the oldest of the winged insects [35]. Odonata individuals have a relatively long life cycle (when compared to mosquitoes and other Diptera [36]), up to a year in the tropics [37]. They are widely distributed in aquatic systems [15,38], and present a dual life cycle, where larvae are aquatic and adults are terrestrial/aerial [39]. The fact that Odonata larvae and adults occupy two different environments suggests that this group can provide valuable information on changes occurring in both aquatic and terrestrial environments [40,41].

In this context, we evaluated the relative importance of environmental and spatial factors in the structuring of the local communities of adult Odonata in both preserved streams and sites altered by farming and ranching activities in two regions of eastern Amazonia, which varied considerably in area and the integrity of the study streams [42]. Our operational hypothesis was that the structure of local communities is determined primarily by environmental factors, such as the structure of the riparian vegetation, human impacts, and physical–chemical variables, in both types of environment (preserved and altered). In this case, as demonstrated in a number of previous studies of aquatic macroinvertebrates [23,43,44,45], the communities will be structured according to the assumptions of niche theory, given the sensitivity and ecological requirements of the different Odonata species. Spatial factors should have little effect on community structure, especially considering the waterway connections and the respective dispersal capacities of the two Odonata suborders.

We do not expect all individuals (e.g., suborders) to respond to these effects in a uniform manner. In the specific case of the Odonata, there is considerable variation in the dispersal capacity and ecological requirements of the two suborders, and even among members of the same suborder (see [29,31,46]). In this case, if the environment is the primary factor structuring the Odonata community, its effects would be observed more clearly in the species of the suborder Zygoptera, which have more restricted environmental requirements and reduced dispersal capacity, related primarily to their smaller body size [47].

## 2. Materials and Methods

### 2.1. Study Areas

**Figure 1:**
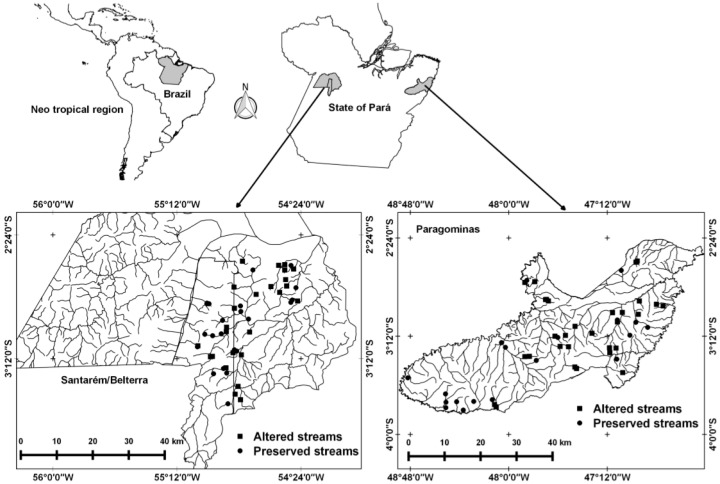
Drainage basins and streams sampled in the regions of Santarém/Belterra and Paragominas, in Brazilian Amazonia, Pará.

The present study focused on two areas of eastern Amazonia, one in the municipality of Paragominas and the other in the municipalities of Santarém and Belterra, all in the Brazilian state of Pará (Figure 1). Paragominas (1.9 Mha) is located in northeastern Pará (02°59′51” S, 47°21′13” W), with mean annual precipitation of 1766 mm, mean annual temperature of 27 °C, and relative humidity of 81% [48]. With a total area of 1 Mha, Santarém (02°26′22” S, 54°41′55” W) and Belterra (02°41′54” S, 54°53′18” W) are located in the western extreme of the state, where mean annual precipitation is 1920 mm, the mean annual temperature is 25 °C, and relative humidity is 86% [49]. The climate in Paragominas is predominantly of the *Af* type in the Köppen classification system, while in Santarém and Belterra, types *Am* and *Amw* predominate (in both municipalities). These climate types correspond to tropical rainy climates with a short, well-defined dry season.

The vegetation of the Paragominas region is classified as dense rainforest [50], while tropical forest is the predominant vegetation type in Santarém and Belterra, with the exception of the Amazonian savannas found in the northeastern extreme of Santarém [51]. Both regions are characterized by a gradient of land use [52], with impacted areas including secondary forests, regrown after clear-cutting [53], plantations of trees, predominantly of eucalypt (*Eucalyptus* sp. L’Hér.), teak (*Tectona grandis* L.), and Brazilian firetree (*Schizolobium parahyba* var. *amazonicum* Huber ex Ducke), cattle pasture, and other crops, mainly rice and soybean (*Oryza sativa* L.; *Glycine max* L.) [31,42,52]. At the opposite extreme, large tracts of well-preserved, climax forest can still be found, with little evidence of logging [31,42,52]. In this context, the inclusion of the two regions in the study amplified both its environmental and spatial dimensions, given the potential for the expansion of the gradient examined.

### 2.2. Data Collection

#### 2.2.1. Biological Samples

A total of 98 streams were sampled in the two regions during the dry season, of which 50 were surveyed in Paragominas between June and August 2011, and 48 in Santarém-Belterra, in July and August 2010. Data were collected during the dry season because the intensity of the precipitation during the rainy season creates logistic difficulties and interferes in the activity patterns of adult Odonata (see [31,38,54,55]). In addition, some studies [56,57] have shown that Odonata abundance and species richness tend to be higher during the dry season and sampling was restricted to these months to reduce ‘noise’ due to seasonal effects [58].

In each stream, a 150 m stretch was demarcated and subdivided into 10 sections of 15 m, separated by margin-to-margin transects, which were denominated A (furthest downstream) through K, furthest upstream [59,60]. The sampling consisted of the longitudinal 15 m sections subdivided into three segments of 5 m, of which only the first two were sampled in each case, resulting in a final sample of 20 five-meter segments.

Temperature and relative humidity were measured in a shaded location adjacent to each stream (150 m stretch). Samples were collected invariably between 10:00 h and 14:00 h, when the sun’s rays reached the main stream channel. These minimum conditions were necessary to guarantee that all the different groups of Odonata (conformers, heliotherms and endotherms) were present at the moment of sample collection (see [54,55,61]). The adult specimens were collected using an entomological handnet (40 cm Ø, 65 cm deep, with an aluminum handle 90 cm long) following the protocol established by Ferreira-Peruquetti and De Marco [62] and Oliveira-Junior et al. [31]. The specimens were preserved following Lencioni’s [63] protocol. Each stream was sampled by a single collector during 1 hour [31], with approximately 3 minutes in each segment. The distance between the sampling points guaranteed the independence of the samples [31]. 

The specimens were identified using taxonomic keys and specialized illustrated guides [63,64,65,66,67,68,69,70], as well as comparisons with the voucher material available in the collection of the Zoology Museum of the Federal University of Pará, Brazil. Once identified, the specimens were deposited as vouchers in the same collection. 

#### 2.2.2. Environmental Predictors 

Nessimian et al. [71] developed the habitat integrity index (HII) to quantify the integrity of streams. This index is made up of 12 items that describe the environmental conditions found in the stream, through a visual evaluation of the following features: land use patterns adjacent to the riparian vegetation, the width of the riparian vegetation and its degree of preservation, the condition of the riparian forest with a radius of 10 m of the stream, the types of sediment found in the channel, and the presence of retention devices, the structure and erosion of the margins, the characteristics of the stream bed in terms of the substrate, aquatic vegetation, detritus, and distribution of areas of rapids, pools, and meanders. Each item is made up of four to six alternatives, which are ranked to evaluate the integrity of the system, ranging from 0 (least integrity) to 1 (most integrity). This index is related directly to the degree of conservation of the environment and has been applied successfully in a number of previous studies that have evaluated the integrity of aquatic systems [14,29,31,72,73]. 

Peck et al.’s [60] environmental assessment protocol, known as the *Field Operations Manual for Wadeable Streams* (FOMWS), and physical-chemical factors of the water were used to measure environmental predictors at each stream, in order to standardize the measurement of environmental conditions. This protocol uses a set of quantitative metrics of the physical habitat, such as the riparian vegetation, human impacts not related to the riparian vegetation, and features of the structure of the stream channel. For each category, the following predictor variables were used: (i) Riparian Vegetation: mean amount of canopy over the channel, canopy of large trees, herbaceous stratum, exposed soil, and mean total cover; (ii) Human Impact: proximity of buildings, roads, refuse dumps, plantations, forestry plantations, and total impact; (iii) Structure of the channel: humid width, height of the seasonal bed, angle of the margins, macrophytes, quantity of woody debris (>60 cm in diameter and 5 m in length) in the channel, hanging vegetation, immersion of the channel, slope, and sinuosity of the stretch of stream, depth and discharge. This protocol includes a set of environmental metrics (e.g., canopy opening, width, depth, and human impact) that have been shown to play an important role in the structuring of Odonata communities. All the metrics included in the protocol were calculated using the procedures described by Kaufmann et al. [59].

A Horiba^®^ U-51 multiparameter probe was used to measure five physical and physical–chemical descriptors of the water: electrical conductivity (μS/cm), pH, dissolved oxygen (mg/L), total dissolved solids, and water temperature (°C). 

#### 2.2.3. Spatial Predictors

Spatial variables were generated using a Principal Coordinates of Neighboring Matrices (PCNM) approach [74]. The PCNM was based on a matrix of Euclidean distances among the sample sites, which was run through a principal coordinates analysis (PCoA), and the eigenvectors with positive eigenvalues were extracted for a posteriori analyses. These eigenvectors, normally known as spatial filters [75], were used as our spatial predictor variables, resulting in a final total of five spatial filters. The PCNM was executed in the SAM (spatial analysis in macroecology) program [76].

### 2.3. Data Analysis 

To avoid problems of multicollinearity among the environmental variables [60] metrics and the physical and physical-chemical variables of the water, matrices of Pearson correlation coefficients were established. When a correlation of at least 0.70 was found between two variables, we selected the variable known to have a systematic influence on the occurrence of Odonata [77]. A forward stepwise procedure was used to select the environmental variables model (a set of 239 variables) which best explain the variation observed in the composition of the Odonata, anisopteran, and zygopteran communities.

To evaluate the distinction between the conservation categories of the streams, the values of the 12 items of the HII that describe the prevailing environmental conditions of the study streams were summarized in a principal components analysis (PCA). To determine which principal components should be retained for analysis, we used the randomness obtained by the broken-stick model [78]. To test whether the conservation categories (preserved and altered) were significantly different from one another, the scores generated by the PCA were tested using Student’s *t*-test (*p* < 0.05). The variation of each conservation category was obtained by a multivariate permutation analysis of dispersal (PERMDISP= *P*_perm_), based on the distance of each sample to the group mean [79].

A partial redundancy analysis, or pRDA [80], was used to evaluate the relative importance of environmental and spatial factors on the structure of the Odonata communities. Prior to this analysis, the density data were transformed using Hellinger’s method [81], which is recommended for the preservation of the Euclidian distance among sample units in an *n*-dimensional space, as well as controlling for the problem of the lack of linearity in the raw abundance data [81,82]. The total variation explained by the pRDA is partitioned between the exclusive [a and b] and combined [c] contributions of the predictors [83]. In this case, the variation in community structure is divided into (i) “pure” environment [a], the portion accounted by environmental factors, without the influence of spatial factors, and (ii) “pure” space [b], the portion of the variation explained exclusively by spatial factors, independently of the environmental factors. The variation explained by a combination of environmental and spatial factors is represented in [c]. An additional, residual fraction [d] is not accounted for by either environmental or spatial factors [83]. This analysis was robust, even in the presence of the collinear variables in the tables of exploratory data, which did not need to be excluded for the tests [80,82].

All the analyses were run in the vegan and MASS packages [84,85] of the R program [86]. 

## 3. Results

### 3.1. Description of the Odonata Communities

A total of 3588 Odonata specimens were collected, representing nine families, 49 genera, and 134 species. Most (2415) of the specimens belonged to the suborder Zygoptera, distributed in six families (Calopterygidae, Coenagrionidae, Dicteriadidae, Megapodagrionidae, Perilestidae, and Polythoridae), 20 genera, and 71 species. The suborder Anisoptera was represented by 1173 specimens, belonging to three families (Aeshnidae, Gomphidae and Libellulidae), 29 genera, and 62 species. 

In the Zygoptera, the Coenagrionidae was the most common family, with 1155 specimens, with species of the genus *Argia* being the most common (n = 438), followed by *Epipleoneura* (n = 351), and *Neoneura* (n = 92). In the case of the Anisoptera, the Libellulidae was the most abundant family, with 1154 specimens, and the genera *Erythrodiplax* (n = 552), *Oligoclada* (n = 167) and *Diastatops* (n = 114) were the most common.

### 3.2. Abiotic Characteristics of the Streams 

The principal variables that accounted for the distinction of the sample points were HII (which varied from 0.15 to 0.99), electrical conductivity (7.00–76.20 µS/cm), mean canopy cover (0.0–100.0%), buildings or infrastructure (0.00–0.63%), total impact (mean of all impact: 0.00–2.73%), discharge (0.01–1.45 m³/s), and depth (5.76–86.89 cm). The association of the two PCS axes represented 46.56% of the environmental variation (model selected for the Odonata).

**Figure 2:**
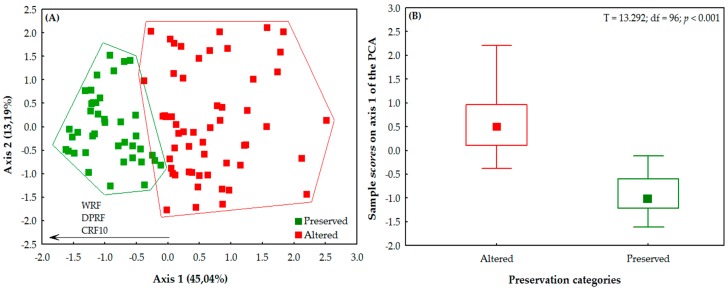
(**A**) Ordination of environmental variables (values of 12 items of the habitat integrity index (HII)); (**B**) relation between the scores of the samples on axis 1 of the ordination (PCA) and conservation (preserved and altered) of the streams surveyed in two regions of Brazilian Amazonia, in the state of Pará. (WRF = width of the riparian forest; DPRF = degree of preservation of the riparian forest; CRF10 = condition of the riparian forest within a radius of 10 m).

Based on the HII variation (0.15 to 0.99), the 98 streams were classified in two arbitrary categories of conservation (Figure 2A): altered (HII = 0.15–0.69; 55 streams) and preserved (0.70–0.99; 43 streams). The separation of the streams into two conservation categories was significant (*t* = 13.292; df = 96; *p* < 0.001) (Figure 2B).

The association of the two PCA axes represented 58.23% of the environmental variation. Only the first axis was analyzed, given that the second axis did not present an observed value greater than that estimated by the broken-stick procedure (which was adopted whenever a situation of this type arose). The first axis explained 45.04% of the results (eigenvalue = 5.40). In this analysis, the samples were clearly separated by conservation category. The preserved streams had a positive relationship with environmental integrity, being grouped in the direction of the highest values for the width and degree of preservation of the riparian forest (Figure 2A). The altered streams were characterized by a significant loss and changes in the state of preservation of the riparian forest, with a group of these streams being associated negatively with the integrity of this vegetation (Table 1, Figure 2A).

It is important to note here that the variables that most contributed to the formation of the first axis are closely related to the physical structure of the riparian vegetation. These variables are associated negatively with the level of conservation of these environments, including the width of the riparian forest (WRF), degree of preservation of the riparian forest (DPRF), and the condition of the riparian forest within a radius of 10 m (CRF10) (Table 1, Figure 2A). 

With regard to their dispersal in relation to the centroid of each group, the conservation categories varied significantly in environmental heterogeneity (F = 16.134; *P*_perm_ = 0.001). Altered streams were more heterogeneous than preserved ones, i.e., altered environments present a mean of 0.23 environmental variation, more than preserved environments.

### 3.3. Effects of Environment and Space on Odonata Communities 

**Figure 3:**
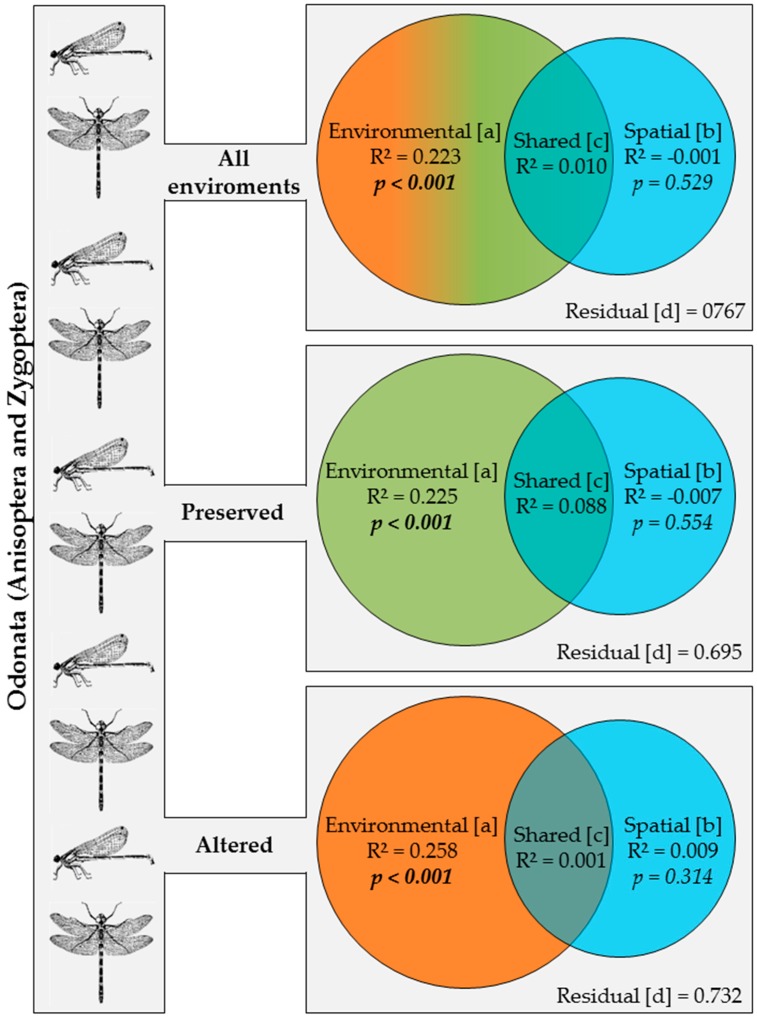
Partition of the variation using the matrix of abundance of the species of Odonata (Anisoptera and Zygoptera) sampled in: all environments; only preserved streams; and only altered streams in two regions of Brazilian Amazonia in the state of Pará. Bold values are significant at the *p* < 0.05 level.

Considering all the environments, 22% of the variance in the composition of the Odonata communities was explained exclusively by environmental factors (fraction [a]), with no effect of spatial factors (fraction [b]) (Figure 3). Considering only the preserved environments, 23% of the variance was attributed exclusively to environmental factors (fraction [a]), while the effect of spatial factors (fraction [b]) was, once again, not significant (Figure 3). Considering only the altered environments, 26% of the variance was explained exclusively by environmental factors (fraction [a]) and 1% was explained solely by spatial factors, i.e., fraction [b] (Figure 3).

**Figure 4:**
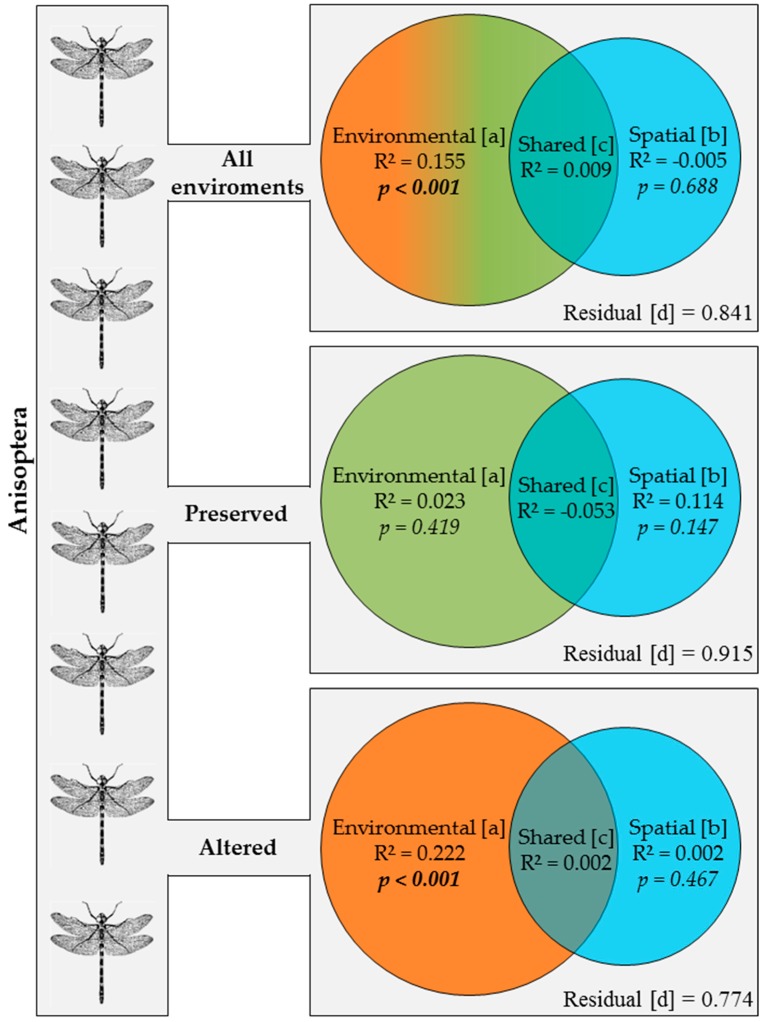
Partition of the variation using the matrix of abundance of the species of Anisoptera sampled in: all environments; only preserved streams; and only altered streams in two regions of Brazilian Amazonia in the state of Pará. Bold values are significant at the *p* < 0.05 level.

In all types of environment, 15% of the variance in the composition of the Anisoptera community was explained solely by environmental factors (fraction [a]), whereas spatial factors (fraction [b]) did not explain any of the variation found in this community (Figure 4). When the preserved environments are considered separately, none of the variance in species composition is explained by environmental or spatial factors, but in the altered environments, approximately 22% of the variance in the composition of the Anisoptera community was explained by environmental factors (fraction [a]), but none was accounted for by the spatial fraction (Figure 4).

**Figure 5:**
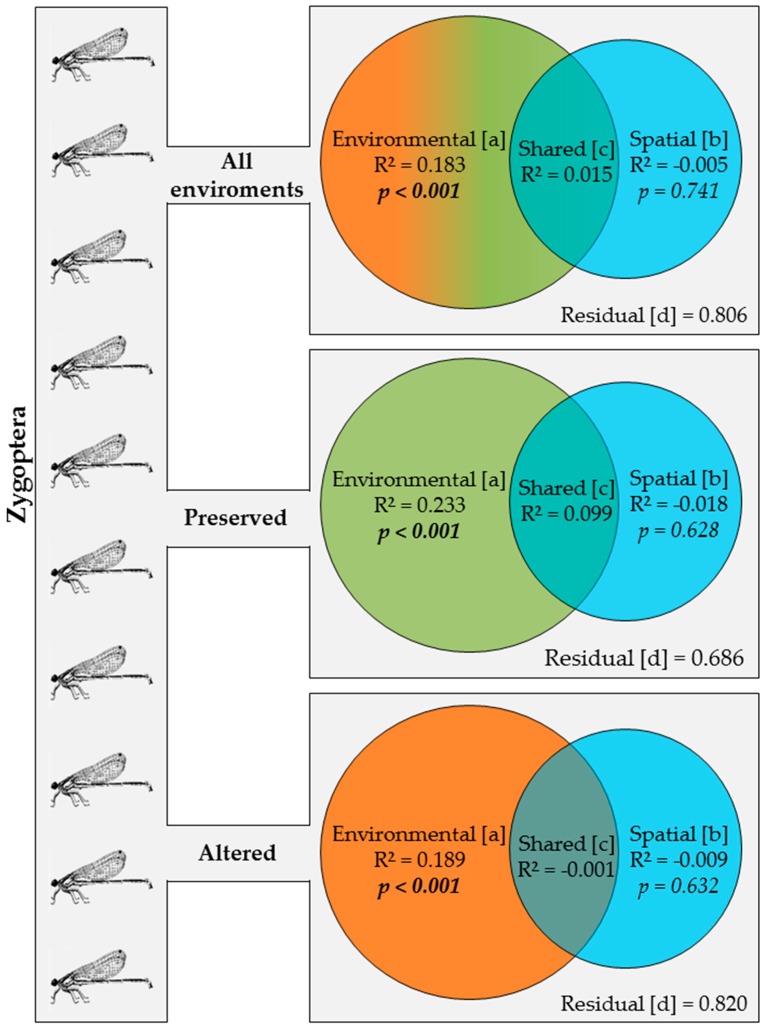
Partition of the variation using the matrix of abundance of the species of Zygoptera sampled in: all environments; only preserved streams; and only altered streams in two regions of Brazilian Amazonia in the state of Pará. Bold values are significant at the *p* < 0.05 level.

In the case of Zygoptera, approximately 18% of the variance in species composition was explained by environmental factors (fraction [a]), whereas none was explained by the spatial fraction [b]. Considering only the preserved environments, 23% of the variance was explained by environmental factors (fraction [a]), once again with no apparent effect of spatial factors (fraction [b]). In the case of the altered environments, approximately 19% of the variance in the composition of the Zygoptera community was explained by environmental factors and, once again, none by spatial factors (Figure 5).

## 4. Discussion

We have demonstrated that the characteristics of Odonata communities in eastern Amazonia are associated closely with the environmental conditions of the aquatic ecosystems, validating our hypothesis that local communities respond primarily to environmental factors, such as the structure of the riparian vegetation, human impacts, and physical–chemical variables.

From a metacommunity perspective, a number of recent studies have shown that local communities are structured by both environmental factors and spatial processes [10,87,88]. However, our results indicate that only environmental factors are important determinants of the variation in the communities (Odonata, Anisoptera, and Zygoptera) in small streams of eastern Amazon.

The structuring of local communities of aquatic insects has often been accounted for by the interaction between spatial processes and local environmental factors [44,45,89], although environmental factors are generally considered the most important [45,90]. The predominance of local environmental factors indicates that the mechanisms predicted by niche theory are more important than those predicted by neutral theory to explain the structuring of the aquatic insect communities of the small streams of eastern Amazon. While some studies have shown that landscape-level factors, such as the geology, area, and other geographic parameters (latitude, distance between sites), are at least as important as environmental factors, such as the physical–chemical characteristics of the water [91,92], the present study indicated that these variables have a reduced power of explanation. Given this, the species-sorting predictive model of distribution of the local Odonata communities may be determined by the heterogeneity of the environment, acting as a filter of the distribution of species according to the adequacy of habitats, according to the availability of the resources required by the different species. In this context, dispersal is an essential determinant of local species composition, given that the species will become established wherever adequate environmental conditions are found [10].

Many metacommunity studies have concluded that a major portion of the variability remains unaccounted for [44,93,94,95]. Streams are highly dynamic ecosystems, with considerable stochastic variation [96], and as we did not consider biotic interactions, a large percentage of the unexplained variability was in fact expected. A large part of this variance may be related to other factors, such as competition and sexual selection, as well as environmental variables not measured here or spatial structures that were overlooked due to their complexity [97].

None of the data sets produced by the present study explained more than 26% of the variability in the composition of the communities (Odonata or Zygoptera and Anisoptera separately) in both types of environment (preserved and altered). This percentage is higher than that recorded by Costa et al. [45] in the ephemeropteran communities in streams in São Paulo, Brazil (17%), or those found in bacteria (11%), phytoplankton (7%), and fish (6%) in streams in Quebec, Canada [98]. Schulz et al. [95] also recorded a percentage of only 14% in phytotelmata in the Atlantic Forest of Brazil. However, Mendes et al. [23] recorded a very similar value (29%) for the influence of environmental factors on the structure of the community of zygopteran larvae in streams of the Brazilian Cerrado. Similar results were also obtained by Heino and Mykrä [43], Costa et al. [45], and Shimano et al. [44], who all showed that the majority of the variability in the composition of ephemeropteran communities was determined by local environmental factors, demonstrating their importance in the structuring of communities of aquatic insects. In streams in central Europe, Feld and Hering [99] observed that 22% of the variation in the communities of benthonic macroinvertebrates was explained by environmental variables.

Many Odonata species are stenotopic, and thus highly dependent on local conditions [100]. In addition, their dispersal mode and mechanisms have a major impact on the organization of the Odonata metacommunities [101]. Given this, the fact that environmental factors have a greater effect on the structure of zygopteran communities, rather than those of anisopterans, may be related to the reduced dispersal capacity of these smaller-bodied Odonata, which are thus more dependent on local conditions (e.g., physical and chemical parameters of the water (conductivity), channel structure, and plant cover), causing them to focus on a specific resource in the environment, in other words, habitat specialists [102]. One good example of a habitat specialist can be found in the genus *Chalcopteryx*—the females of this taxon require a specific type of habitat in which to lay their eggs, and are thus found only in preserved environments [103]. A number of other studies of Odonata communities have also found associations between some species and a given type of habitat [18,104,105]. In streams in Greece, local environmental variables also played a more important role in the variation in the heteropteran community than geographic dispersal, which was also attributed to the reduced dispersal capacity of this group [106].

Zygopteran species are sensitive to environmental variation due to the restrictions of their ecophysiological requirements [4]. In many cases, the amount of forest cover, especially that located within a radius of 400 m of the body of water, will have a negative effect (when absent) on the occurrence of species with adults of small size, such as members of the genera *Telebasis* and *Oxyagrivern* [107]. As thermal conformers, these species have a high degree of thermal conductance associated with their body size, and body temperature will vary according to the amount of sunlight found in the environment [23,108]. In tropical environments, most zygopteran species are associated with shady areas, given their high surface to volume ratio of their bodies, which makes them vulnerable to overheating and dehydration under constant exposure to sunlight [4,109]. In this context, areas with more open vegetation may represent barriers to the effective dispersal of zygopterans [27,47,109].

Even though anisopterans are more tolerant of different environmental conditions [102], environmental variables still have a fundamental influence on community structure when considering all the environments surveyed or only the altered ones. In preserved environments, however, neither environmental nor spatial effects were observed in the composition of the anisopteran community. Larger individuals, which are typical of this suborder, tend to have more efficient homeostatic mechanisms and greater mobility, which allows them to be more tolerant of different environmental conditions and to range over greater distances [102]. Changes in the landscape may be contributing to an increase in the number of more opportunistic anisopterans (e.g., *Diastatops*), reducing the effects of both environmental and spatial components on the structuring of this suborder.

Good dispersers, like many anisopterans, should not be limited in any major way by dispersal effects, and when significant spatial structuring of community composition is found, it will usually be related to mass effects [110]. In general, a closer relationship will be found between the dissimilarity of communities and geographic distance over a greater spatial scale [111]. Reduced spatial effects may mean that even areas more than 500 kilometers apart—as in the present study—are not sufficiently distant from one another for the detection of the effects of limitations on dispersal [112,113]. In Finland, Astorga et al. [114] found that freshwater organisms are controlled more by environmental factors than by dispersal, even at distances of up to 1100 km.

## 5. Conclusions

As the two Odonata suborders responded in distinct ways to the two factors (environmental and spatial), the independent analysis of each suborder would probably be an effective measure for the improvement of biomonitoring studies. We suggest that studies of this types would have a greater explanatory potential with the addition of other variables related to biotic interactions (e.g., competition), as well as a finer scale of environmental variation. In this context, further studies will be necessary to determine how the Odonata fauna of the streams of the Amazon region behave from this viewpoint.

Given the current biodiversity crisis, the enormous biological diversity found in aquatic ecosystems, and the social and economic value of the preservation of the quality of aquatic resources, it is increasingly important to develop reliable methods for the measurement, monitoring, and eventual mediation of environmental impacts. The Odonata can potentially provide important insights into the conditions of aquatic ecosystems, although these organisms have been widely overlooked.

## Figures and Tables

**Table 1:** The 12 items of the habitat integrity index (HII) that describe the environmental conditions of the study streams sampled in two regions of Brazilian Amazonia in the state of Pará, and their correlations with axes 1 and 2 of the principal components analysis (PCA).

Variables of the Habitat Integrity Index (HII)	Loading	
	Axis 1	Axis 2
Pattern of land use in the area outside the riparian vegetation zone (PLUORV)	0.028	0.087
Width of the riparian forest (WRF)	0.141 *	0.033
Degree of preservation of the riparian forest (DPRF)	0.133 *	0.013
Condition of the riparian forest within a radius of 10 m (CRF10)	0.133 *	0.012
Retention devices (RD)	0.079	0.059
Sediments in the channel (SC)	0.083	0.104
Structure of the river bank (SRB)	0.038	0.231
Excavation under bank (EUB)	0.081	0.040
River bed (RB)	0.008	0.262
Areas of rapids, pools or meanders (ARPM)	0.067	0.100
Aquatic vegetation (AV)	0.085	0.058
Detritus (D)	0.123	0.001
Eigenvalue	5.405	1.582
Broken-stick	5.404	6.987

* Variables that most contributed to the formation of axis 1 (≥0.13).
